# Severe monkeypox-virus infection in undiagnosed advanced HIV infection

**DOI:** 10.1007/s15010-022-01901-z

**Published:** 2022-08-15

**Authors:** Christoph Boesecke, Malte B. Monin, Kathrin van Bremen, Stefan Schlabe, Christian Hoffmann

**Affiliations:** 1 Department of Medicine I, Bonn University Hospital, Venusberg-Campus 1, 53127 Bonn, Germany; 2 German Centre for Infection Research (DZIF), Partner-site Cologne-Bonn, Cologne, Germany; 3 Infektionsmedizinisches Zentrum Hamburg, Hamburg, Germany; 4 University Hospital of Schleswig-Holstein, Campus Kiel, Kiel, Germany

**Keywords:** HIV, Monkeypox, MPX, Immunodeficiency, AIDS

A 40-year old male presented to his GP with a red spot on the tip of his nose which was initially classified as a sunburn. Within three days, the nasal area progressed to necrosis (Fig. 1). In parallel, typical MPXV lesions (confirmed by PCR) appeared on the whole body with serious infection of the penis and oral mucosa. The patient was transferred to a tertiary care hospital for tecovirimat treatment. Diagnostic work-up revealed a concomitant syphilis of longer duration (TPPA 1:2560, VDRL 1:8) and an advanced HIV infection with a CD4 *T* cell count of 127/uL. The patient had never been tested for sexually transmitted diseases (STD) before. The patient was treated with oral tecovirimat 600 mg bid for 7 days in addition to antiretroviral therapy (bictegravir/emtricitabine/tenofoviralafenamide single tablet p.o. qd) for the HIV infection and ceftriaxone 2 g i.v. for 10 days for the syphilis. The monkeypox lesions on the skin dried out and the nose partially improved with less swelling. Most cases of MPXV infection so far have been reported as mild and controlled HIV infection does not appear to be a risk factor for severe courses [1–4]. However, this case illustrates the potential severity of MPXV infection in the setting of severe immunosuppression and untreated HIV infection.

**Fig. 1:**
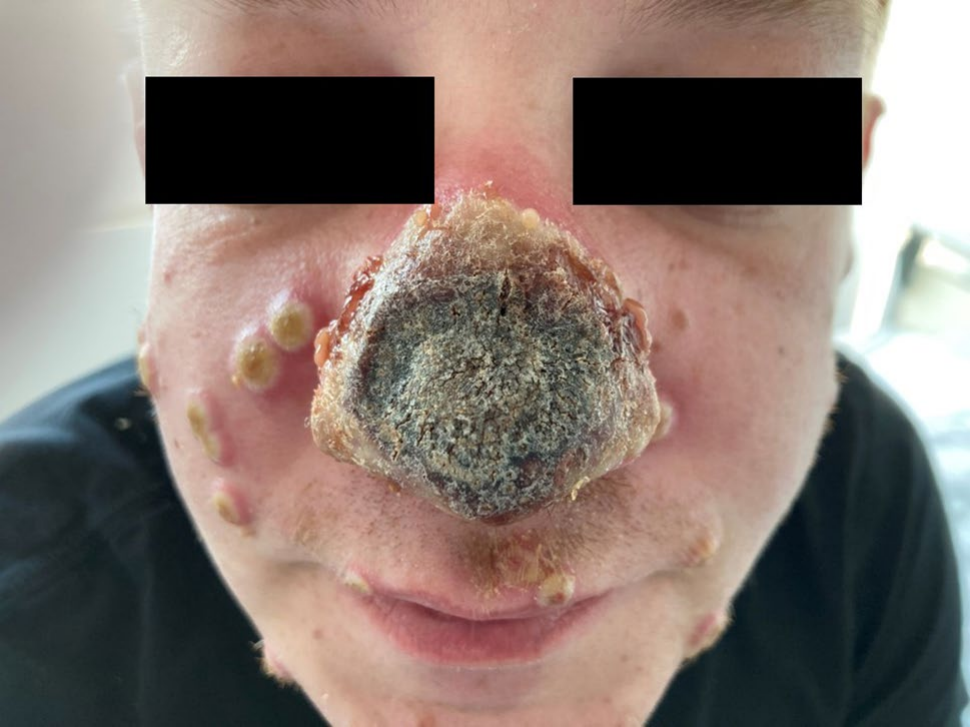
Nasal necrosis and skin lesions due to MPXV infection in a patient with advanced undiagnosed HIV infection

## Data Availability

Upon request from corresponding author.
